# Pathological mechanisms of type 1 diabetes in children: investigation of the exosomal protein expression profile

**DOI:** 10.3389/fendo.2023.1271929

**Published:** 2023-10-11

**Authors:** Baoling Bai, Kang Gao, Kexin Zhang, Lingyun Liu, Xiaobo Chen, Qin Zhang

**Affiliations:** ^1^ Beijing Municipal Key Laboratory of Child Development and Nutriomics, Capital Institute of Pediatrics, Beijing, China; ^2^ Endocrinology Department, Children’s Hospital of Capital Institute of Pediatrics, Beijing, China

**Keywords:** type 1 diabetes, exosome, proteomics, TMT6, insulin treatment

## Abstract

**Introduction:**

Type 1 diabetes (T1D) is a serious autoimmune disease with high morbidity and mortality. Early diagnosis and treatment remain unsatisfactory. While the potential for development of T1D biomarkers in circulating exosomes has attracted interest, progress has been limited. This study endeavors to explore the molecular dynamics of plasma exosome proteins in pediatric T1D patients and potential mechanisms correlated with T1D progression

**Methods:**

Liquid chromatography-tandem mass spectrometry with tandem mass tag (TMT)6 labeling was used to quantify exosomal protein expression profiles in 12 healthy controls and 24 T1D patients stratified by age (≤ 6 years old and > 6 years old) and glycated hemoglobin (HbA1c) levels (> 7% or > 7%). Integrated bioinformatics analysis was employed to decipher the functions of differentially expressed proteins, and Western blotting was used for validation of selected proteins' expression levels.

**Results:**

We identified 1035 differentially expressed proteins (fold change > 1.3) between the T1D patients and healthy controls: 558 in those ≤ 6-year-old and 588 in those > 6-year-old. In those who reached an HbA1c level < 7% following 3 or more months of insulin therapy, the expression levels of most altered proteins in both T1D age groups returned to levels comparable to those in the healthy control group. Bioinformatics analysis revealed that differentially expressed exosome proteins are primarily related to immune function, hemostasis, cellular stress responses, and matrix organization. Western blotting confirmed the alterations in RAB40A, SEMA6D, COL6A5, and TTR proteins.

**Discussion:**

This study delivers valuable insights into the fundamental molecular mechanisms contributing to T1D pathology. Moreover, it proposes potential therapeutic targets for improved T1D management.

## Introduction

Destruction of β cells by auto-reactive CD8^+^ T cells in the pancreas leads to the development of type 1 diabetes (T1D) (1, 2), a devastating chronic autoimmune disease that ultimately results in chronic hyperglycemia and a need for lifelong insulin therapy. The number of children with T1D in 2021 exceeded 1.1 million worldwide, and the incidence rate is growing by nearly 3% per year (3, 4). In China, the annual incidence of childhood T1D is about 2.02–5.3 per 100,000 person-years (5). About one-quarter to one-third of children with newly diagnosed T1D present with diabetic ketoacidosis, and diabetic coma is the most common cause of death in younger patients (6, 7). Additionally, macro- and microvascular complications, such as atherosclerosis and cardiovascular disease, are the main cause of excess morbidity and mortality in T1D patients (8, 9). Prevention of T1D and its complications remains the most effective means of reducing the morbidity, premature mortality, and related family distress in this population.

While the age of onset of childhood T1D varies between individuals, T1D can develop at any age. Children typically experience the onset of T1D symptoms during one of two peak age periods: preschool years (~4–6 years) and adolescence (~10–14 years) (10–12). Currently, the most common approach to treating diabetes is to regulate glycemia. Glycosylated hemoglobin (HbA1c) measurements, which approximate a person’s average blood glucose level over the previous 3 months, are often used to guide therapy (13, 14). The current glycemic target in T1D is an HbA1c level < 7.0% (< 53 mmol/mol) (15), which aims to minimize complications without an unacceptable risk of hypoglycemia (16). However, pediatric T1D is a heterogeneous disorder in which clinical presentation and disease progression may vary considerably (17). Registry data revealed that only a minority of children with T1D from 20 countries or regions achieved the target; that is, among 44,058 people aged < 15 years, the proportions with an HbA1c level < 7.5% (58 mmol/mol) varied from 15.7% to 46.4% (18). These data suggest a need for further development in the diagnosis and management of T1D.

Exosomes of 40–100 nm in diameter, which are small membranous vesicles of endocytic origin from the endosomal system, serve as multifactorial mediators of cell-to-cell communication and metabolic regulation via their microRNA, DNA, or protein cargo (19, 20). Exosomes are present in every biological fluid, including plasma/serum, and are influenced by multiple physiological and pathological conditions, including those in T1D disease (21, 22); thus, they have potential as diagnostic and prognostic markers for T1D (23). Additionally, exosomes can be used to improve angiogenesis, stimulate immunomodulatory actions, and boost the success rate of islet transplantation, showcasing their therapeutic potential for T1D (24). While a 2022 study by Diaz Lozano et al. clearly established the value of proteomic profiling of whole plasma and plasma-derived extracellular vesicles in pre-diabetic non-obese diabetic mice (an experimental model of T1D) (22), such profiling of human T1D samples has not been reported.

In this study, we used TMT6-labeled quantitative proteomics to analyze protein cargo in plasma exosomes from pediatric patients with T1D. We investigated potential differences in proteomic profiles between age groups and whether these differences were affected by insulin therapy. Additionally, we employed bioinformatics analysis to explore the functions of differentially expressed proteins (DEPs) between the patients and healthy controls, deepening our understanding of the pathological mechanisms of T1D.

## Results

### Clinical characteristics of children with T1D

**Figure 1:**
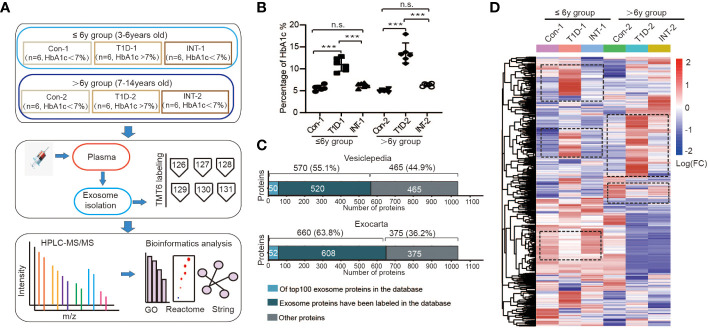
Plasma exosomal protein expression profiles in pediatric T1D patients. **(A)** Schematic representations of the age groupings and subgroupings of healthy controls and T1D patients, TMT6 quantification, and bioinformatics analysis workflow. **(B)** Levels of HbA1c in each subgroup, n.s. to indicate *P*>0.05 and *** to indicate *P* ≤ 0.001. **(C)** Comparison of the acquired protein expression profiles of T1D exosomes with the Vesiclepedia and Exocarta exosome database. **(D)** Heatmap of protein expression levels across different subgroups.

Pediatric patients with T1D were initially divided into two age groups: ≤ 6-year-old and > 6-year-old. Two control subgroups (Con1/2; n = 6 each) of healthy volunteers matched for age, sex, body mass index (BMI), and ethnicity were also included (**Table 1**). The detailed experimental design is illustrated in **Figure 1A**. The two age groups showed no significant differences in age of T1D diagnosis, BMI, sex, and systolic or diastolic blood pressure. Additionally, the time from symptom onset to T1D diagnosis between the two age groups or within the subgroups were comparable, and none of the patients had complications, such as kidney or eye damage. Given that genetic factors may play an important role in T1D development, we noted that 5 out of 12 T1D patients aged ≤ 6 year-old and 6 out of 12 T1D patients aged > 6 year-old had a family history of diabetes.

**Table 1:** Baseline characteristics of the participants in the study.

	≤6y group			>6y group		
Subgroups	Con-1	T1D-1	INT-1	Con-2	T1D-2	INT-2
No. of participants	6	6	6	6	6	6
Geographical location	Northern Chinese	Northern Chinese	Northern Chinese	Northern Chinese	Northern Chinese	Northern Chinese
Time from symptom onset to T1D diagnosis, month	–	0.43 ± 0.08	1.62 ± 0.60	–	1.41 ± 0.44	0.63 ± 0.26
T1D diagnosed age, y	–	3.50 ± 0.50	4.20 ± 0.58	–	8.45 ± 1.08	6.33 ± 0.88
Age, y	4.50 ± 0.43	4.33 ± 0.42	5.16 ± 0.40	8.50 ± 0.56	9.17 ± 0.65	9.17 ± 0.48
Males/Females, (n)	3/3	3/3	3/3	3/3	3/3	3/3
BMI, kg/m2	15.36 ± 0.37	15.85 ± 0.88	15.37 ± 0.60	15.19 ± 0.44	14.81 ± 0.81	15.33 ± 0.67
Glycosylated hemoglobin, (%HbA1c)	5.63 ± 0.24**	10.95 ± 0.60	6.22 ± 0.21**	5.02 ± 0.13##	13.60 ± 0.94	6.30 ± 0.15##
Systolic blood pressure (SBP), mmHg	98.60 ± 0.87	97.00 ± 2.08	100.50 ± 1.26	100.00 ± 0.82	103.80 ± 2.58	101.50 ± 2.22
Diastolic blood pressure (DBP), mmHg	60.00 ± 2.09	63.00 ± 1.91	66.00 ± 4.32	64.00 ± 2.83	63.75 ± 2.39	60.67 ± 1.33
History of heredity with diabete, n(%)	0	2 (33%)	3 (50%)	0	3 (50%)	3 (50%)
Complication, n	0	0	0	0	0	0

Values are mean ± SEM.

** In the ≤6 years old group, comparing T1D-1 with Con-1 or INT-1, P<0.01.

## In the>6 years old group, comparing T1D-2 with Con-2 or INT-2, P<0.01.

Each age group was then further categorized into two subgroups on the basis of HbA1c% status: T1D-1/2 (HbA1c% > 7.0%; n = 6) and INT-1/2 (HbA1c% < 7.0% following ≥ 3-mo insulin treatment; n = 6). Among the ≤ 6-year-old subgroups, there was a mild difference in HbA1c% values between the Con-1 (5.63 ± 0.24%) and INT-1 (6.22 ± 0.21; *P* = 0.312) subgroups, but both were significantly lower than that of the T1D-1 subgroup (10.95 ± 0.60; *P* < 0.001). Among the > 6-year-old subgroups, the HbA1c% values were comparable between the Con-2 (5.02 ± 0.13%) and INT-2 (6.30 ± 0.15%; *P* = 0.122) subgroups; again, both were significantly lower than that of the T1D-2 subgroup (13.60 ± 0.94; *P* < 0.001) (**Figure 1B**).

### Global exosomal proteome analysis in children with T1D

To improve the detection and quantification of exosomal proteins, two TMT6-labeled replicates were produced (**Supplementary Figure 1**). Totally, we identified 1868 proteins across all six subgroups, listed in **Table S1**. After removing duplicates and unnamed proteins, we cross-referenced the remaining 1035 proteins using exosomal protein database of Vesiclepedia and Exocarta, respectively (25, 26). There were 570 (55.1%) or 660 (63.8%) overlapping proteins, which included known exosome markers CD9 and heat shock protein (HSP)70 (27, 28), and 50/52 proteins were among the top 100 proteins identified in exosomal preparations, confirming the successful isolation and identification of exosomes. We also detected other unannotated proteins, comprising immunoglobulins, complement factors, lipoproteins, and other exosomal proteins, with potential specificity for pediatric T1D (**Figure 1C**).

Cluster analysis was also performed on the 1035 known proteins, revealing significant variation in the expression patterns among the subgroups in the two age groups, as depicted by the cluster areas within dotted black boxes in **Figure 1D**. These results suggested that the protein expression patterns in T1D are age-specific.

### Expression patterns of the exosomal proteome in the ≤ 6-year-old groups

**Figure 2:**
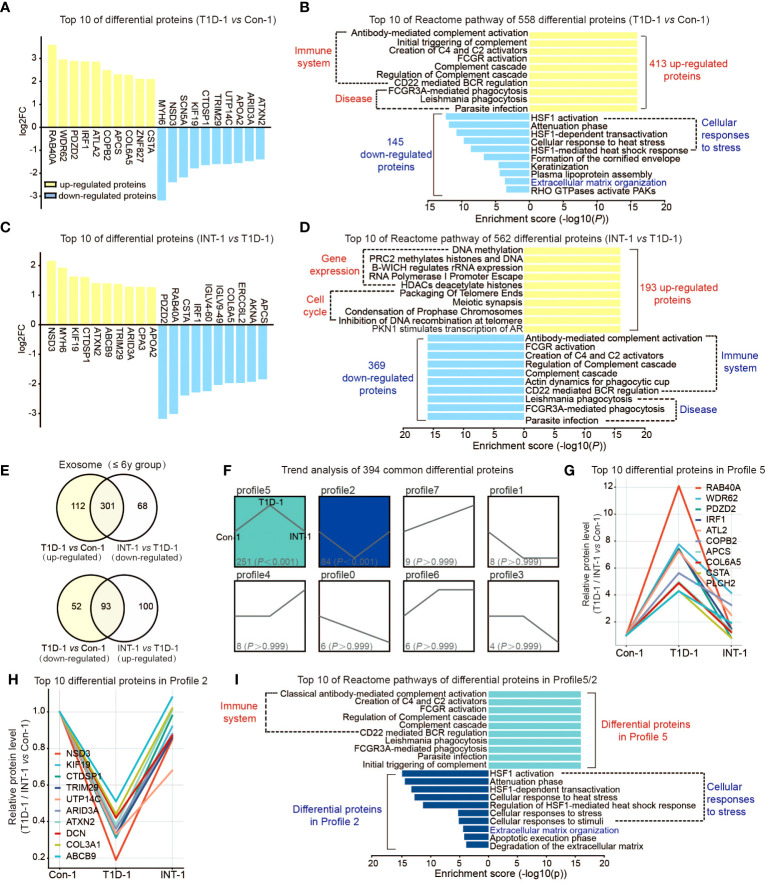
Protein expression profiles of plasma exosomes in pediatric T1D patients aged ≤ 6 years old. **(A)** Top 10 upregulated/downregulated proteins in the T1D-1 and Con-1 subgroups. **(B)** Reactome pathway enrichment analysis of 413 upregulated and 145 downregulated exosomal proteins in T1D-1 patients, showing the top 10 enriched pathways. **(C)** Top 10 upregulated/downregulated proteins in the INT-1 and T1D-1 subgroups. **(D)** Reactome pathway enrichment analysis of 193 upregulated and 369 downregulated exosomal proteins in INT-1 patients, showing the top 10 enriched pathways. **(E)** Venn diagram comparing all DEPs between T1D-1 *vs.* CON-1 and INT-1 *vs.* T1D-1. **(F)** Expression trends of the 394 DEPs from the two above comparisons shared across subgroups. **(G, H)** Top 10 proteins enriched in Profiles 5 and 2. **(I)** Reactome pathway enrichment analysis of proteins in Profiles 5 and 2, illustrating the enriched signaling pathways.

First, we compared exosomal proteome expression in the ≤ 6-year groups. Compared to Con-1, T1D-1 showed differential expression of 558 proteins (413 upregulated and 145 downregulated). The top 10 were RAB40A, WDR62, and COL6A5, etc (**Figure 2A**). Upregulated proteins were related to the immune system, and downregulated proteins were linked to the cellular stress response and extracellular matrix (ECM) organization (**Figure 2B**).

Next, we found 562 DEPs identified in comparison of INT-1 and T1D-1, which were apparently sensitivity to insulin treatment, including RAB40A, PDZD2, CSTA, IRF1, and COL6A5 (**Figure 2C**). Downregulated proteins were enriched in the immune system, while upregulated proteins were enriched in gene expression regulation (as the epigenetic regulations: DNA methylation, histone methylation and acetylation regulation, etc.) and cell cycle pathways (as the packaging of telomeres during cell division) (**Figure 2D**), suggesting increased cell cycle activation after insulin treatment.

Venn analysis of the aforementioned comparisons revealed 164 (112 + 52) proteins that were expressed in T1D-1 but not in INT-1, indicating that they were insulin treatment-insensitive. Insulin treatment appeared to induce 168 (68 + 100) new DEPs identified in INT-1, which were mainly enriched in the cell cycle and gene expression regulation (**Figure 2E**, **Supplemental Figures 2C, D**). Additionally, compared to Con-1, of the 413 up-regulated proteins found in T1D-1, 301 were down-regulated in INT-1. Similarly, of the 145 down-regulated proteins detected in T1D-1, 93 were up-regulated in INT-1. In total, these two comparisons share 394 (301 + 93) DEPs. Trend analysis of these proteins revealed that patients with HbA1c > 7% showed enhanced expression of 251 proteins (Profile 5: the higher of the protein expression level, the more severe of the disease) and decreased expression of 84 proteins (Profile 2: the lower the protein expression level, the more severe the disease). However, these changes in protein expression were mitigated control of HbA1c at < 7% (i.e., the INT-1 subgroup) (**Figure 2F**). **Figures 2G, H** illustrates the top 10 DEPs in Profiles 5 and 2, respectively. Furthermore, reactome pathway analysis indicated that these proteins were mainly enriched in pathways involving the immune system, cellular stress response, and ECM (**Figure 2I**).

### Expression patterns of the exosomal proteome in the > 6-year-old groups

**Figure 3:**
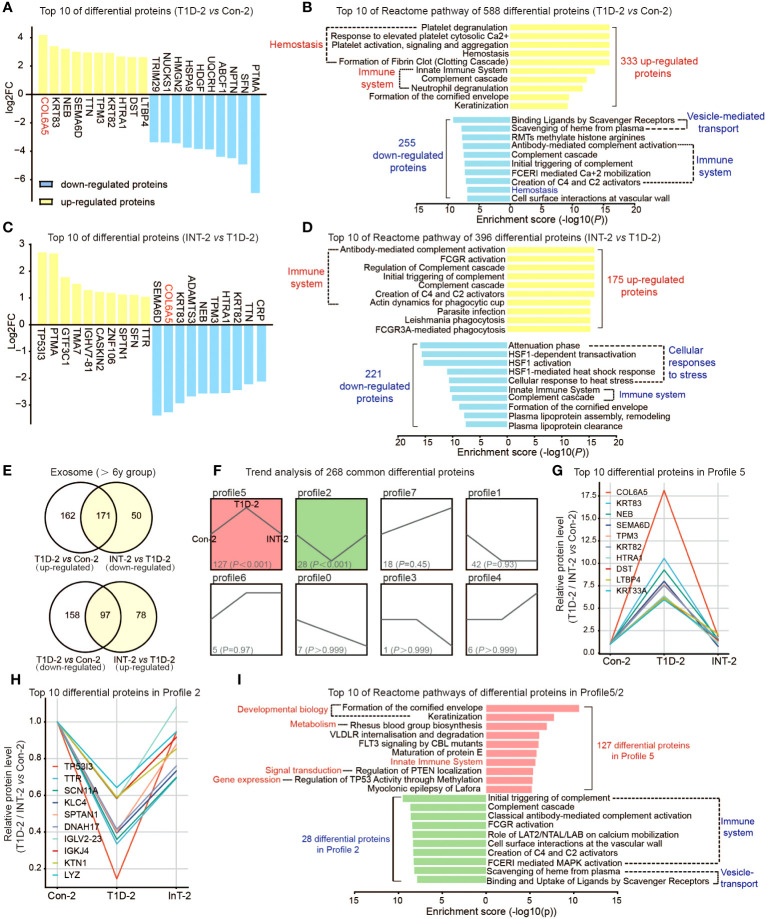
Protein expression profiles of plasma exosomes in pediatric T1D-2 patients aged > 6 years old. **(A)** Top 10 upregulated/downregulated proteins in the T1D-2 and Con-2 subgroups **(B)** Reactome pathway enrichment analysis of 333 upregulated and 255 downregulated exosomal proteins in T1D-2 patients, showing the top 10 enriched pathways. **(C)** Top 10 upregulated/downregulated proteins in INT-2 patients compared to T1D-2. **(D)** Reactome pathway enrichment analysis of 175 upregulated and 221 downregulated exosomal proteins in INT-2 patients, showing the top 10 enriched pathways. **(E)** Venn diagram comparing all DEPs between T1D-2 *vs.* CON-2 and INT-2 *vs.* T1D-2. **(F)** Expression trends of the 268 DEPs from the two above comparisons shared across subgroups. **(G, H)** Top 10 proteins enriched in Profiles 5 and 2. **(I)** Reactome pathway enrichment analysis of proteins in Profiles 5 and 2, illustrating the enriched signaling pathways.

Similar to the **≤** 6-year group, we initially compared T1D-2 with Con-2, which identified 588 DEPs, comprising 333 upregulated and 255 downregulated proteins in T1D-2. The top DEPs included COL6A5, KRT83, NEB, and TRIM29 (**Figure 3A**). Pathway analysis suggested that these DEPs were mainly enriched in hemostasis and immune system pathways (**Figure 3B**).

We further compared INT-2 with T1D-2, noting 396 DEPs. Proteins such as COL6A5, KRT83, NEB, SEMA6D, and TPM3 were apparently sensitive to insulin treatment, and were predominantly enriched in immune system and cellular stress response pathways (**Figures 3C, D**).

Combining the two aforementioned comparisons (**Figure 3E**), we identified 320 (162 + 158) proteins that were expressed in T1D-2 but not in INT-2, and were mostly enriched in homeostasis, ECM, gene expression, and signal transduction. There were 128 (50 + 78) proteins with specific differential expression in INT-2, suggesting that changes in their expression were triggered by insulin treatment. These DEPs were primarily enriched in the immune system, cellular stress response, vesicle-regulated transport, and disease (**Supplemental Figures 3C, D**).

Finally, we focused our analysis on the 268 (171 + 97) DEPs identified in both comparisons. Trend analysis indicated that nearly half (127 of 268) exhibited expression patterns belonging to Profile 5, whereas 28 belonged to Profile 2 (**Figure 3F**). **Figures 3G, H** illustrates the top 10 proteins in Profiles 5 and 2, respectively, which included COL6A5, KRT83, NEB, SEMA6D, TP53I3, and TTR. Furthermore, altered proteins in Profiles 5 and 2 were predominantly enriched in pathways involving the immune system, developmental processes, and signal transduction (**Figure 3I**). Overall, these results highlighted the role of differential proteins and associated pathways in the insulin therapy response and T1D pathophysiology in patients over 6 years of age.

### Comparison of DEPs in plasma exosomes of the two age groups

**Figure 4:**
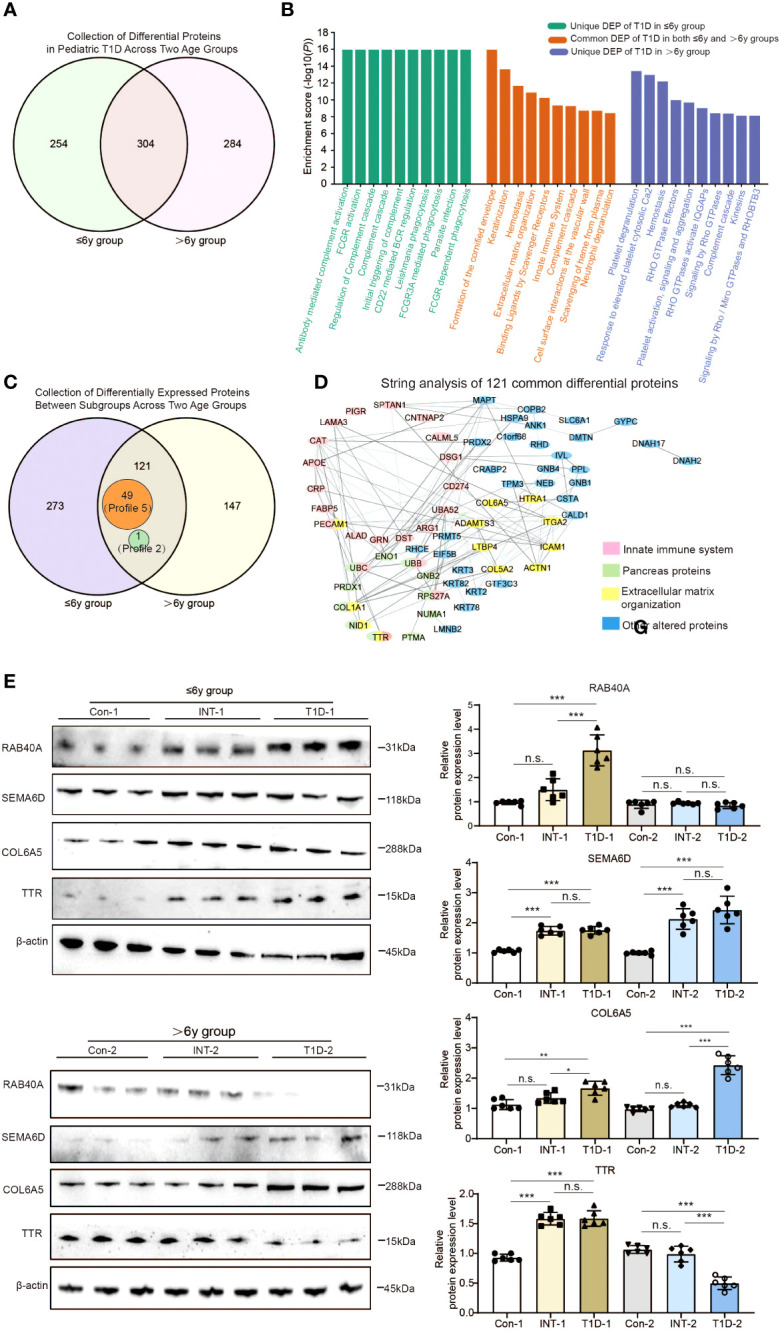
Comparison of exosomal protein expression profiles in the two age groups of pediatric T1D patients. **(A)** Venn analysis of DEPs between T1D patients and healthy controls in both age groups. **(B)** Reactome pathway enrichment analysis of proteins that were differentially expressed: only in the ≤ 6-year-old T1D-1 group; only in the > 6-year-old T1D-2 group; and in both age groups of T1D patients. **(C)** Venn analysis showing 121 proteins with differential expression across both age groups and all subgroups, which included 49 Profile 5 proteins and 1 Profile 2 protein. **(D)** STRING analysis of the protein-protein interactions among the 121 proteins. **(E)** Western blotting to validate the presence of the four alter proteins s with clinical relevance in both age groups of T1D patients. n.s. to indicate *P*>0.05, * to indicate *P* < 0.05, ** to indicate *P* ≤ 0.01 and *** to indicate *P* ≤ 0.001.

We conducted a Venn analysis to determine whether there were common or unique protein expression patterns in the two age groups. Among the 558 DEPs identified in the comparison of T1D-1 *vs* Con-1 in the ≤ 6-year-old and the 588 DEPs identified in the comparison of T1D-2 *vs* Con-2 in the > 6-year-old group, there were 304 overlapping proteins (**Figure 4A**). Furthermore, 254 proteins showed age-specific differential expression only in the ≤ 6-year-old T1D-1, whereas 284 proteins showed age-specific differential expression only in the > 6-year-old group. Pathway analysis of these three sets of DEPs showed that they were mainly enriched in pathways involved in hemostasis, ECM organization, vesicle-mediated transport, and the innate immune system (**Figure 4B**). Additionally, Gene Ontology (GO) annotation revealed that the cellular components, biological processes, and molecular functions enriched by the DEPs were similar between the ≤ 6-year-old and > 6-year-old T1D subgroups (**Supplemental Figures 2A, B**, **3A, B**). This implied that, although the protein expression patterns varied, the underlying biological pathways and functional roles may be similar between the two age ranges.

We also performed a simultaneous analysis of the 394 DEPs identified in both comparisons of ≤ 6-year-old subgroups and the 268 DEPs identified in both comparisons of > 6-year-old subgroups (**Figures 2E**, **3E**, **4C**). This revealed that there were 273 DEPs identified in both comparisons of ≤ 6-year subgroups that did not show differential expression in the > 6-year-old subgroups. One such protein was RAB40A. Conversely, there were 147 such proteins unique to the > 6-year-old subgroups, which included SEMA6D. The differing expression patterns of these proteins might be due to children with T1D exhibit age-specific, spatiotemporal protein expression at two different developmental stages. However, there were also 121 DEPs identified in both comparisons of the two age groups (**Figure 4C**, **Supplemental Figure 4**). Of these, 49 proteins (Profile 5) showed upregulation in the T1D group patients and downregulation in the INT group patients (e.g. COL6A5), suggesting that their differential expression between T1D subgroups may be age-independent. Another 71 proteins exhibited distinct patterns, such as TTR, which was upregulated in T1D-1 and downregulated in INT-1 in ≤ 6-year-old patients, but downregulated in T1D-2 and upregulated in INT-2 in > 6-year-old patients.

Functional analysis of these 121 proteins using Kyoto Encyclopedia of Genes and Genomes (KEGG) pathway and STRING analyses identified 11 altered proteins enriched in the pancreas (false discovery rate < 0.001), 21 linked to the innate immune system, and 12 belonging to ECM components (**Figure 4D**). Among these, TTR is known to not only originate from the pancreas, but also to participate in the immune system and contribute to ECM establishment, the multifunctionality of TTR suggests that it plays a crucial role in the progression of T1D.

We validated the data with western blotting of RAB40A, SEMA6D, COL6A5, and TTR, which were chosen on the basis of their differential expression in the dataset, assay feasibility, and T1D relevance (**Figure 4E**). RAB40A was highly expressed only in the ≤ 6-year-old T1D-1 subgroup, and it returned to levels comparable to Con-1 and INT-1. Conversely, SEMA6D was overexpressed in both age groups’ T1D-1/2 subgroups, however, insulin treatment cannot effectively reduce its protein expression levels to a degree similar to the Con-l/2 subgroup. Both age groups showed elevated COL6A5 expression levels in the T1D-1/2 subgroups that returned to near-normal levels in the INT-1/2 subgroups. Differences in TTR were evident between the subgroups, with distinct regulatory patterns in each subgroup (**Figure 4E**). These results were mostly aligned with the quantitative TMT6-labeling outcomes.

### Comparison with previous exosomal proteome studies of T1D patients in different age groups

Finally, we compared our data with findings from several serum proteome studies in T1D patients (29). **Table 2** shows the variance in protein expression patterns across these studies. For instance, compared to healthy controls, adiponectin (ADIPOQ) was highly expressed in sera of 1139 T1D patients aged 0~90 years (30), but downregulated in 13 children with T1D aged 2~12 years (31). Our study found that ADIPOQ was upregulated in the ≤ 6-year-old T1D-1 subgroup and downregulated in the > 6-year-old T1D-2 subgroup, suggesting age-dependent variation in expression among our pediatric T1D subgroups, potentially reconciling the discrepancies across previous studies.

**Table 2:** Protein candidate biomarkers discovered by proteomics.

Gene symbol	Protein name	Regulation (T1D/Con)	Regulation (previously reported)	Function
ADIPOQ	Adiponectin	≤6y, up; >6y, down	Zhi et al (30), up* Moudler et al (31), down*	Glucose homeostasis
APOA4	Apolipoprotein A-IV	≤6y, no change; >6y, no change	Zhi et al (30), up von Tonerne, et al (32), down Moudler et al (31), down	Leukocyte adhesion
APOC4	Apolipoprotein C-IV	≤6y, no change; >6y, no change	von Tonerne, et al (32), down Moudler et al (31), down	Viral infection
APOE	Apolipoprotein E	≤6y, down; >6y, down	von Tonerne, et al (32), down	is essential for the normal catabolism of triglyceride-rich lipoprotein constituents
APOM	Apolipoprotein M	≤6y, no change; >6y, up	von Tonerne, et al (32), up	involved in lipid transport
ATXN2	Ataxin-2	≤6y, down; >6y, down	von Tonerne, et al (32), down	regulate RNA processing and nutrient receptor internalization and metabolism
AZGP1	a-2-glycoprotein 1 (zinc)	≤6y, up; >6y, up	Metz et al (33), up Zhang et al (34), down	Lipid mobilization activity
BTD	biotinidase	≤6y, up; >6y, up	von Tonerne, et al (32), up	biotinyl transferase activity
C3	Complement C3b	≤6y, no change; >6y, down	Zhi et al (30), down von Tonerne, et al (32), down Zhang et al (34), down	Innate immunity, complement activation
C8B	complement C8 beta chain	≤6y, up; >6y, up	von Tonerne, et al (32), up	mediates cell lysis, and it initiates membrane penetration of the complex
CRP	C-reactive protein	≤6y, up; >6y, up	Zhi et al (30), up	Proinflammatory
FN1	fibronectin 1	≤6y, no change; >6y, no change	von Tonerne, et al (32), down	involved in cell adhesion and migration processes
GSN	gelsolin	≤6y, down (mild); >6y, down (mild)	Zhang et al (34), down	functions in both assembly and disassembly of actin filaments
IGFBP2	insulin-like growth factor binding protein 2	≤6y, no change; >6y, up	Zhi et al (30), up	binds IGF-I and IGF-II with high affinity, or it can remain intracellular, interacting with many different ligands
ITIH1	inter-alpha-trypsin inhibitor heavy chain 1	≤6y, down; >6y, down	von Tonerne, et al (32), down	implicated in multiple inflammatory diseases
KNG1	Kininogen 1 isoform 1	≤6y, no change; >6y, no change	Zhi et al (30), up von Tonerne, et al (32), down Zhang et al (34), up	Innate immunity, DC activation
LUM	Lumican	≤6y, no change; >6y, no change	Metz et al (33), up	regulate collagen fibril organization and circumferential growth, corneal transparency, and epithelial cell migration and tissue repair
PA2G4	Lymphocyte activation/proliferation erbB3 binding protein	≤6y, no change; >6y, down	Zhi et al (30), down	Cell proliferation
PGLYRP2	peptidoglycan recognition protein 2	≤6y, down (mild); >6y, down (mild)	Zhang et al (34), down	play a scavenger role by digesting biologically active peptidoglycan into biologically inactive fragments
PPBP	platelet basic protein	≤6y, up >6y, up (mild)	Zhang et al (34), up	stimulate various cellular processes including DNA synthesis, mitosis, glycolysis, intracellular cAMP accumulation, etc.
SAA2-SAA4	serum amyloid A	≤6y, no change; >6y, up	Zhi et al (30), up	associated with chronic inflammatory diseases
SERPINA6	Corticosteroid-binding protein	≤6y, no change; >6y, up	Metz et al (33), up Zhang et al (34), up	Correlate to insulin deficiency or response
SERPINF2	Alpha-2-antiplasmin	≤6y, no change; >6y, up	von Tonerne, et al (32), down Zhang et al (34), up	Serine protease inhibiton
SERPING1	plasma protease C1 inhibitor	≤6y, up (mild); >6y, up (mild)	Zhang et al (34), up	involved in the regulation of the complement cascade
TGFBI	transforming growth factor beta induced	≤6y, no change; >6y, no change	Zhi et al (30), down	Autoimmunity
TPM4	tropomyosin 4	≤6y, no change; >6y, up	Zhi et al (30), down	Endothelial function
TTR	transthyretin	≤6y, up; >6y, down	von Tonerne, et al (32), down Zhang et al (34), down	Involve in β-cell stimulus- secretion coupling

* Up/down indicates that the protein is significantly up/down regulated in serum of patients with diabetes relative to the healthy controls.

Other proteins, such as APOA4, APOC4, FN1, KNG1, LUM, and TGFBI, showed no differences between the pediatric age groups here, but were differentially expressed in other T1D studies (31, 32). The expression patterns of APOE, APOM, ATXN2, BTD, CRP, GSN, ITIH1, PGLYRP2, and PPBP in both of our T1D age groups were consistent with other studies (33, 34). In other studies, TTR was downregulated in islet autoantibody-positive children (median age, 3.2 years) (32) and ~20-year-old T1D patients (34). In our study, compared to healthy controls, TTR was upregulated in the ≤ 6-year-old T1D-1 subgroup but downregulated in the > 6-year-old T1D-2 subgroup, indicating age-related variation. The other three exosomal DEPs that were examined in this study, namely RAB40A, SEMA6D, and COL6A5, have not been previously reported.

## Discussion

In this study, mass spectrometry combined with quantitative TMT6 labeling technology was utilized to analyze the plasma exosome proteomic profiles of children with T1D aged ≤ 6-year-old and > 6-year-old. While DEPs between healthy controls and T1D patients were mainly enriched in immune pathways, cellular stress responses, cellular homeostasis, and ECM structures, there were differences between the two age groups. In patients with insulin treatment-controlled glycosylated protein levels < 7%, some DEPs returned to normal levels. Our study also identified four potential therapeutic target proteins that could be significant for the treatment of T1D: RAB40A, SEMA6D, TTR and COL6A5. Targeting these proteins may represent a new direction for future drug development and improve our understanding of the impact of age on the pathogenesis of T1D.

In this study, we discovered that many of altered proteins identified in pediatric T1D patients were associated with the immune system, which was consistent with the consensus that T1D is an autoimmune disease (3, 4). Notably, the cellular stress response was also found to participate in T1D among the ≤ 6-year-old patients. In the > 6-year age group, a disturbance in homeostasis appeared to be closely related to T1D onset (**Figures 2B**, **3B**). Following insulin therapy, there were apparent changes in both the immune system and cellular stress response. However, it is noteworthy that changes related to cell cycle and gene expression regulation have been observed in the INT-1 subgroup (≤6-year-old) (**Figures 2D**, **3D**), suggesting their sensitivity to insulin. This finding partial aligns with some previous research reports, where histone methylation was closely associated with the regeneration of pancreatic β-cells, while DNA methylation of the insulin (gene) was significantly linked to the death of β cells (35, 36). Overall, these results suggested that, while all children with T1D suffer from immune system dysregulation, there may be distinct dominant signaling pathways within different age groups. In other words, different sets of alterd proteins are involved in the formation of T1D and the insulin-mediated control of blood glucose.

Regarding specific proteins patterns, both age groups exhibited similarities and differences. For example, 254 proteins showed differential expression in the T1D-1 subgroup (≤ 6-year-old), but not in the T1D-2 subgroup (> 6-year-old), whereas 284 proteins showed differential expression in T1D-2 but not in T1D-1, illustrating developmental stage-specificity during the onset of T1D (**Figures 4A, B**). Still, over half (304) proteins overlapped in both T1D age groups, suggesting that they might be involved in the development of T1D through common signaling pathways, such as the immune system, cellular homeostasis, cellular stress response, and ECM. Age-specific differences were also observed among those with insulin treatment. Overall, for the > 6-year-old group, less than one-third of the 588 DEPs (155: 127 + 28) in T1D-2 showed alleviated expression after insulin treatment. In contrast, this ratio was approximately doubled for the 558 DEPs (335: 251 + 84) in the ≤ 6-year-old group, indicating that, as age increases, the likelihood that a T1D-related DEP will be corrected appears to decrease. Understanding these differences in protein expression patterns may provide valuable insights into the underlying pathogenesis of T1D that lead to the identification of potential therapeutic targets and development of personalized treatment strategies for children with T1D at various developmental stages. Furthermore, our study highlights the importance of considering age-specific characteristics, such as the differential expression and response patterns of RAB40A and SEMA6D observed in this study, when designing T1D research studies and developing therapeutic interventions. We anticipate that this knowledge will contribute to the development of more effective treatments targeted to specific age groups of T1D patients.

Ras-related protein Rab-40A (RAB40A) is a small GTPase and a member of the RAB family within the Ras superfamily (37). RAB proteins typically play regulatory roles in intracellular vesicular transport processes, vesicle biogenesis, vesicle positioning, and protein transport between the endoplasmic reticulum and Golgi apparatus. There are no reports of RAB40A being directly associated with T1D in the literature. However, research has suggested the general involvement of GTPases in glucose homeostasis within the pancreas (38), highlighting the potential relevance of GTPases such as RAB40A in processes related to glucose regulation and pancreatic function. Further research is needed to understand the specific role, if any, that RAB40A plays in T1D or glucose homeostasis.

Semaphorin-6D (SEMA6D) belongs to the semaphorin family of proteins, playing a crucial role in vascular formation, immune regulation, and organogenesis. Sema6d was highly enriched in the endothelial cells of the pancreatic tissue in mouse embryos, suggesting that it may have potential impacts on the development and morphogenesis of the pancreas (39). In addition, mutations at its gene locus have been found to be closely associated with the occurrence of diabetic nephropathy (40), implying that SEMA6D may play a significant role in the onset and progression of diabetes.

COL6A5, which belongs to the type VI collagen family, is a major component of the pancreatic ECM (41). The ECM in islets of Langerhans is critically important for maintaining the healthy state of pancreatic β-cells, and for recognizing and reverting the physiopathology of diabetes (42). However, further research is needed to elucidate any potential association between COL6A5 and T1D.

Transthyretin (TTR) is a protein primarily synthesized in the liver and, to a lesser extent, in the choroid plexus of the brain. TTR functions as a transport protein, responsible for carrying thyroxine (a thyroid hormone) and retinol-binding protein bound to vitamin A in the bloodstream. TTR also plays a crucial role in maintaining normal thyroid hormone and vitamin A levels, which are essential for various physiological processes. There is limited evidence directly linking TTR with T1D. However, some studies have observed changes in TTR levels in individuals with T1D (32, 34), suggesting that it could be involved in the metabolic, inflammatory, or hormonal changes that occur in T1D patients.

In conclusion, we discovered four candidate protein biomarkers of T1D. However, the data obtained in this work cannot be used to determine whether the four candidate biomarkers are predictive or diagnostic of T1D. Furthermore, because this study utilized a relatively low number of biological replicates (n = 6) for each condition, higher numbers of control and T1D samples are required to validate our results in subsequent studies. Once validated, the functions of the candidate biomarkers can be further explored in children with T1D.

## Materials and methods

### Human subjects

A total of 24 T1D patients, were recruited from Capital institute of pediatrics affiliated children’s hospital, and a total of 12 nondiabetic controls were recruited. The diagnostic criteria for T1D was according to the “Standardized Diagnosis and Management of Type 1 Diabetes in Chinese Children (2020)” (43): a fasting blood glucose >7.0 mmol/L; a glucose tolerance test >11.1 mmol/L; HbA1c% >7% (two tests, one day apart). In addition, positive of serum pancreatic autoantibodies and C-peptide levels (<0.3 mmol/L) and considered clinical features such as increased thirst, frequent urination, unexplained weight loss, or blurred vision for a comprehensive diagnosis of T1D (44). The INT sub-group refers to T1D patients whose HbA1c% levels have dropped to < 7% after ≥ 3-months insulin treatment, T1D patients who might not effectively regulate their HbA1c% level to < 7% consequent to irregular insulin usage or other physiological conditions were not included in our study; It’s noteworthy that there were no significant differences in HbA1c% between the T1D-1/2 patients and pre-insulin-treatment INT-1/2 patient. The selection of controls was random; they were outpatients from nondiabetic unit who came to the hospital for health check-ups, and were matched for age, gender and ethnicity. They reported no symptoms such as fever and the exclusion criteria included subjects who had conditions of bones, kidneys, liver, endocrinopathies, other autoimmune diseases or melanoma – all diseases that might influence vitamin metabolism. Specifically, the age range for all children in the ≤ 6 year-old group was from 3 to 6 years old; the age range for all children in the >6 year-old group was from 7 to 14 years old.

### Collection and isolation of plasma exosome samples

All human subject samples were fasted 12h beforehand, about 2 mL blood was harvested via venipuncture using a syringe pre-coated with EDTA. Then centrifuged at 4°C, 3000 x g, for 30 minutes to remove platelets and cell debris to obtain the supernatant, namely plasma. Using this method, we were able to obtain up to 1mL of plasma. The obtained plasma were then aliquoted and stored at -80°C before further processing and/or analyses. Care was taken to avoid hemolysis and if a plasma sample was hemolyzed it was not used in the study. Add 50µl of 1X PBS to 100µl of clarified plasma, followed by the addition of 30µl of exosome precipitation reagent (4484450, Thermofisher). Vortex mix the mixture and incubate at room temperature for 10 minutes. After that, centrifuge at 10000 × g for 5 minutes at room temperature. Aspirate the supernatant with a pipette and discard it, add 1X PBS to the pellet, once the pellets are resuspended, the exosomes are ready for downstream analysis.

### Preparation of serum tryptic digests and TMT6 labeling

For each subgroup, the extracted exosomes from the six individuals were pooled.Samples were enzymatically digested with trypsin before analysis. ∼100 µg of exosomal proteins underwent denaturation with 8 M urea and reduction with 10 mM dithiothreitol at 37°C for 1 h. Cysteine residues were alkylated with 40 mM iodoacetamide in the dark for 1 h at 37°C, then desalted using PD-10 columns. Trypsin (1:50 enzyme-to-protein ratio) was added, followed by a 3 h incubation at 37°C. All steps were performed using a Thermomixer R (Eppendorf) at 800 rpm. Digested samples were cleaned using C-18 SPE tubes (Discovery DSC-18, Supelco), eluted peptides were concentrated via speedvac. Dried peptides were resuspended and was quantified using the Quantitative Colorimetric Peptide Assay (Thermofisher) in triplicate. 50ug of peptide were labeled with TMT6 reagents in a 30% final concentration of acetonitrile for an hour followed by 15 minutes of quenching with 5% hydroxylamine.Then the peptide mixtures were subjected to high-pH, one-dimensional reversed-phase chromatography separation on a Dionex Ultimate 3000 HPLC system. The trypsin digests were injected onto a Phenomenex column (Gemini NX 3U C18 110A; 150 x 2.00 mm) at a flow rate of 200 μl/min. Fractions were collected every 1.5 minutes throughout the run. Subsequently, all 40 collected fractions were combined into 10 fractions, lyophilized, and stored at -80°C for further nano-HPLC/MS/MS analysis.

### Mass spectrometry data analysis

Samples were run on a QExactive HF mass spectrometer (Thermo Scientific, Bremen, Germany) equipped with an UltiMate 3000 RSLC nano System (Dionex, Germering, Germany). MSI data were collected in the Orbitrap at 120k resolution (m/z range 350-1350) with a 100 ms maximum injection time. Charge states 2-6 were sequenced, using a 60s dynamic exclusion window and excluding isotopes. MS2 sequencing occurred in the ion trap after quadrupole selection and CID fragmentation, with an m/z window of 400-2000.The obtained raw data were processed using Peaks X studio (Version 10.0, BSI) by searching a Homosapiens database (www.uniprot.org, UP00005610). Precursor mass error tolerance was set to 10 ppm and fragment mass error tolerance to 0.02 Da. Peptide spectral matches were validated at a 1% FDR using a percolator based on q-values. Post-translational modifications and chemical labeling settings were as followes: fixed carbamidomethylation of cysteine residues and TMT-6plex; variable methionine oxidation and protein N-term acetylation. Protein unique peptides were set to >1, and a high confidence score of -10IgP >20 indicated accurately identified proteins.

### Bioinformatics analysis

Gene ontology (GO) term annotations of differentially abundant proteins in exosome isolated from children T1D was performed using Blast2GO (version 2.6.4) with the default annotation parameters (BLAST e-value threshold of 1e-06, Gene Ontology annotation threshold of 55). Reactome pathway annotation was performed using the Server (https://reactome.org/, Version 84 released on March 29, 2023). Trend analysis was performed by Short Time-series Expression Miner (STEM) software, the correlation coefficient between the expression pattern of protein and the trend it belongs to should be greater than 0.7, after Bonferroni correction, if the adjusted *P*-value is less than 0.05, we consider the enrichment of this trend to be significant (45).

### Western blotting

The exosomal protein samples underwent separation via a 4-12% SDS-PAGE and were then transferred onto polyvinylidene fluoride membranes. The membranes were blocked with 10% skim milk for one hour and incubated overnight at 4 °C with primary antibodies, which included RAB40A (bs-8366R, Bioss, China), SEMA6D (bs-2943R, Bioss), COL6A5 (bs-11047R, Bioss), and TTR (#29872, CST). Following this, the membranes were rinsed using Tris Buffered Saline with Tween 20 before being incubated with infrared-labeled anti-rabbit/mouse IgG antibody. Ultimately, the protein bands were detected using the Image Lab system (Gel Image System, Tanon, Shanghai, China), with their expression levels determined relative to the staining of β-actin (#3700, CST).

### Statistical analysis

The data were analyzed using the SPSS, version 23. Normative distribution of data was tested by P-Plot. All data were normally distributed. Student t-test was used to two‐group comparison. ANOVA, followed by an appropriate post‐hoc analysis, was employed in multiple‐group (>2 groups) comparisons. *P* value less than 0.05 was considered statistically significant.

## Data availability statement

The mass spectrometry proteomics data have been deposited to the ProteomeXchange Consortium (http://proteomecentral.proteomexchange.org) via the iProX partner repository (39) with the dataset identifier PXD043146.

## Ethics statement

The study was approved by Capital institute of pediatrics Ethical Committee (SHERLL2023052). Consent was obtained from each patient. The studies were conducted in accordance with the local legislation and institutional requirements. Written informed consent for participation in this study was provided by the participants’ legal guardians/next of kin.

## Author contributions

BB: Conceptualization, Data curation, Formal Analysis, Methodology, Writing – original draft. KG: Resources, Writing – original draft. KZ: Data curation, Methodology, Software, Writing – original draft. LL: Investigation, Methodology, Validation, Writing – original draft. XC: Funding acquisition, Resources, Supervision, Writing – review & editing. QZ: Funding acquisition, Validation, Visualization, Writing – review & editing.

## Supplementary material

Supplementary Figure 1
**(A)** The number of proteins quantitatively identified by two rounds of TMT6 labeling. **(B)** Box plots of the channel intensity distributions of all 6 labels in the second replicated experiment. The horizontal dashed line represents the set threshold for reporter ion intensity. A plot displaying the average distribution for all labels is also included, which represents the quality control.

Supplementary Figure 2
**(A, B)** GO annotations for altered proteins in T1D-1 and INT-1, respectively; **(C, D)** Reactome pathway enrichment analysis for DEPs specific to T1D-1 or INT-1, respectively.

Supplementary Figure 3
**(A, B)** GO annotations for altered proteins in T1D-2 and INT-2, respectively; **(C, D)** Reactome pathway enrichment analysis for proteins specific to T1D-2 or INT-2, respectively.

Supplementary Figure 4
**(A)** Combined analysis of intergroup proteins (belonging to Profile 5) across the two age groups. **(B)** Combined analysis of intergroup proteins (belonging to Profile 2) across the two age groups.
