# Supplementary figures and images for: Pathological mechanisms of type 1 diabetes in children: investigation of the exosomal protein expression profile
Source: Front Endocrinol (Lausanne). 2023 Oct 11;14:1271929. doi: 10.3389/fendo.2023.1271929 (PMC10599151; doi:10.3389/fendo.2023.1271929)

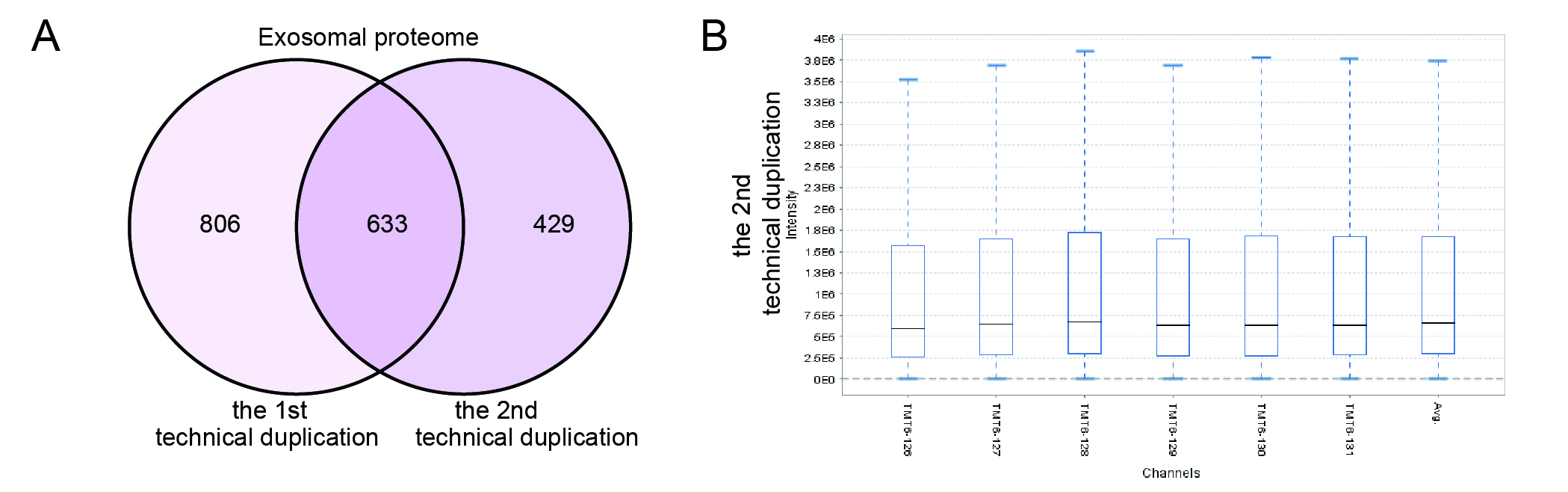

Supplement: Supplementary Figure 1 — (A) The number of proteins quantitatively identified by two rounds of TMT6 labeling. (B) Box plots of the channel intensity distributions of all 6 labels in the second replicated experiment. The horizontal dashed line represents the set threshold for reporter ion intensity. A plot displaying the average distribution for all labels is also included, which represents the quality control. [file Image_1.jpeg]

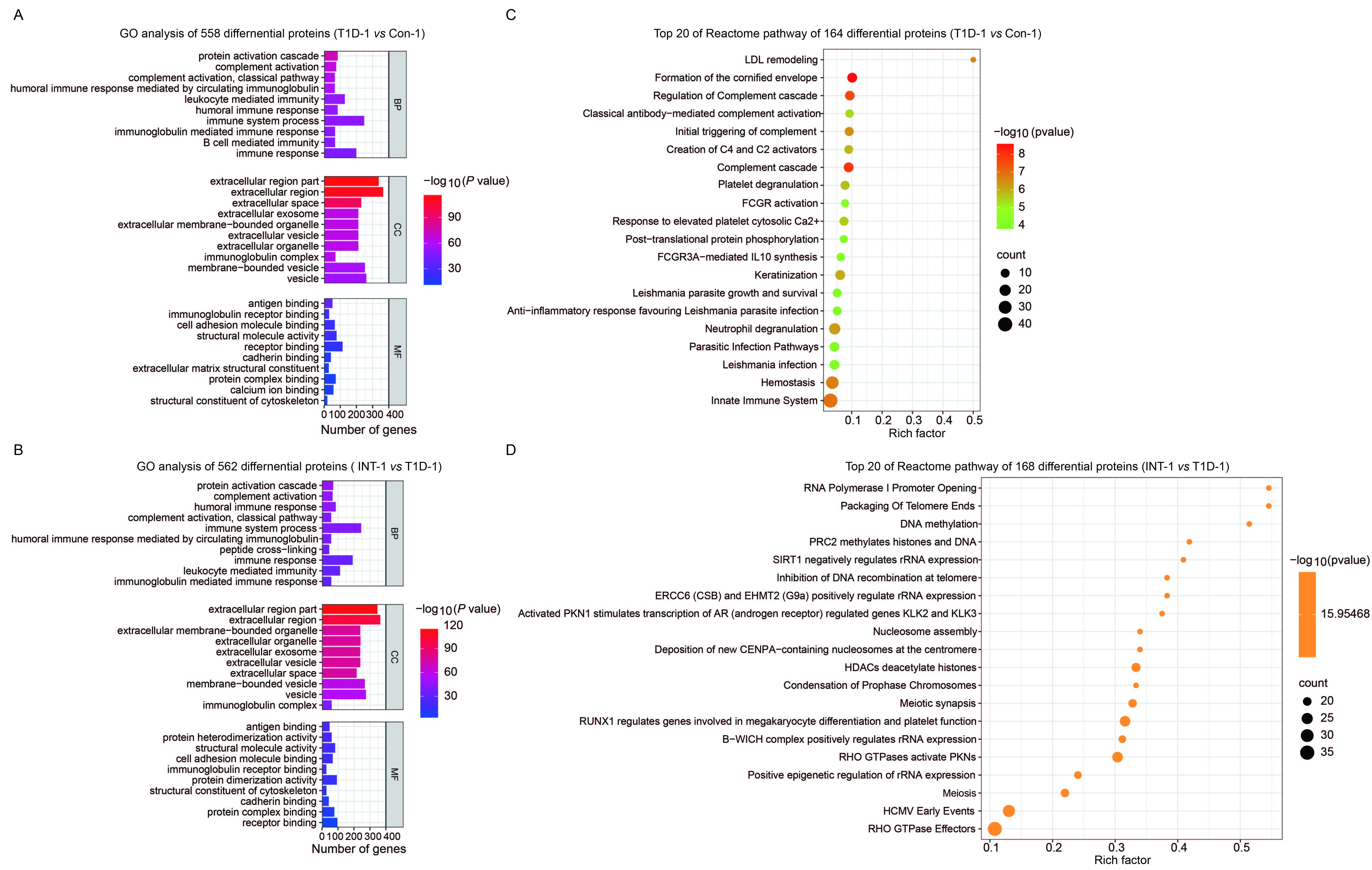

Supplement: Supplementary Figure 2 — (A, B) GO annotations for altered proteins in T1D-1 and INT-1, respectively; (C, D) Reactome pathway enrichment analysis for DEPs specific to T1D-1 or INT-1, respectively. [file Image_2.jpeg]

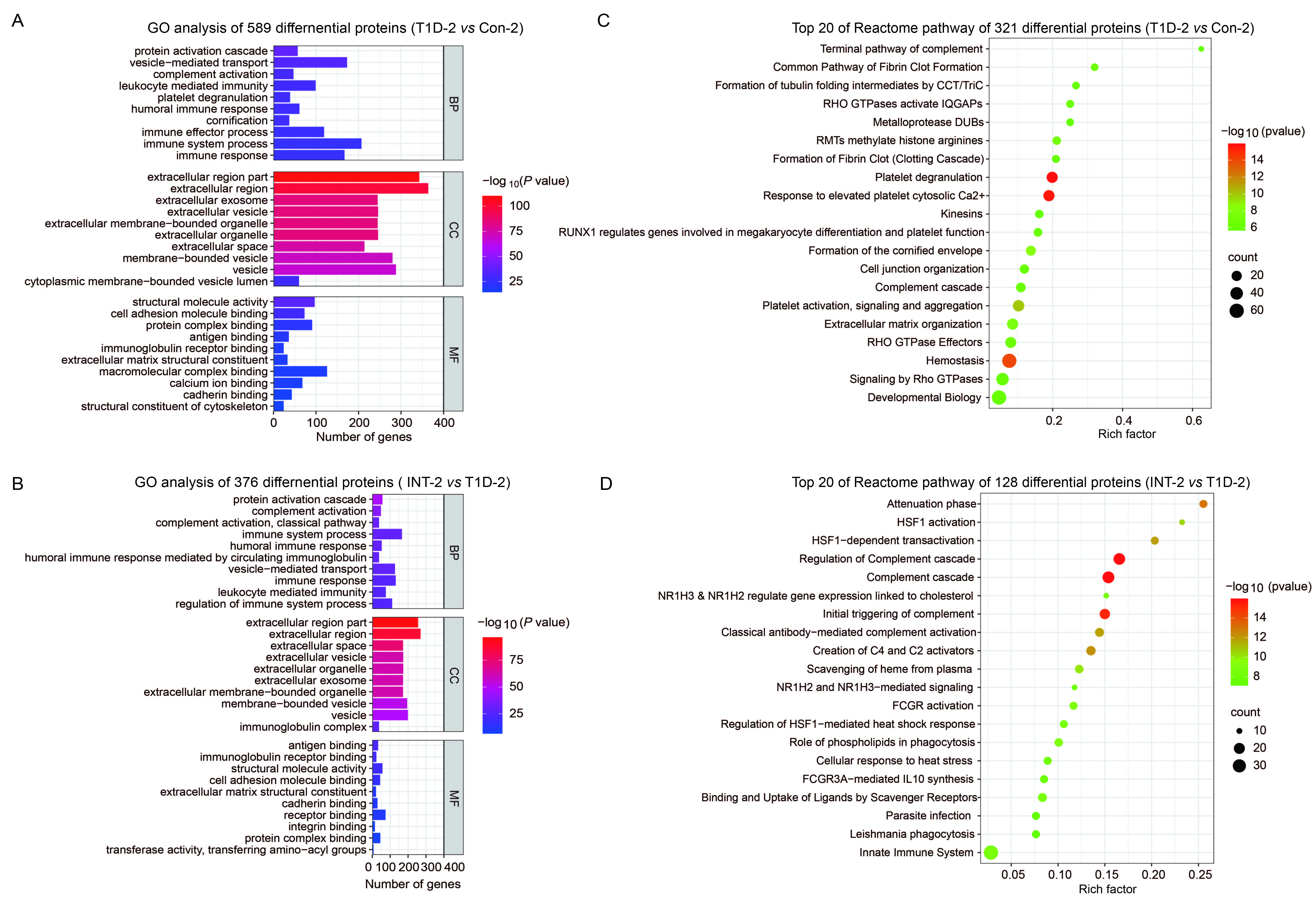

Supplement: Supplementary Figure 3 — (A, B) GO annotations for altered proteins in T1D-2 and INT-2, respectively; (C, D) Reactome pathway enrichment analysis for proteins specific to T1D-2 or INT-2, respectively. [file Image_3.jpeg]

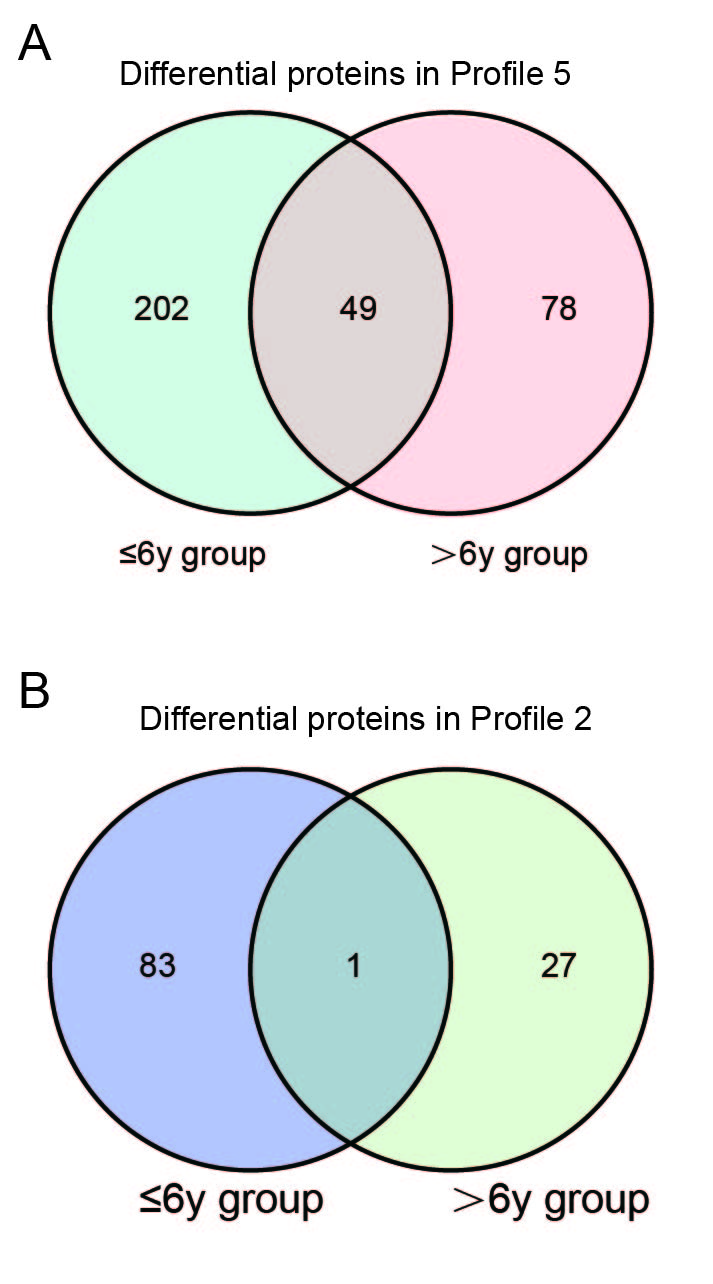

Supplement: Supplementary Figure 4 — (A) Combined analysis of intergroup proteins (belonging to Profile 5) across the two age groups. (B) Combined analysis of intergroup proteins (belonging to Profile 2) across the two age groups. [file Image_4.jpeg]

Figure 4E

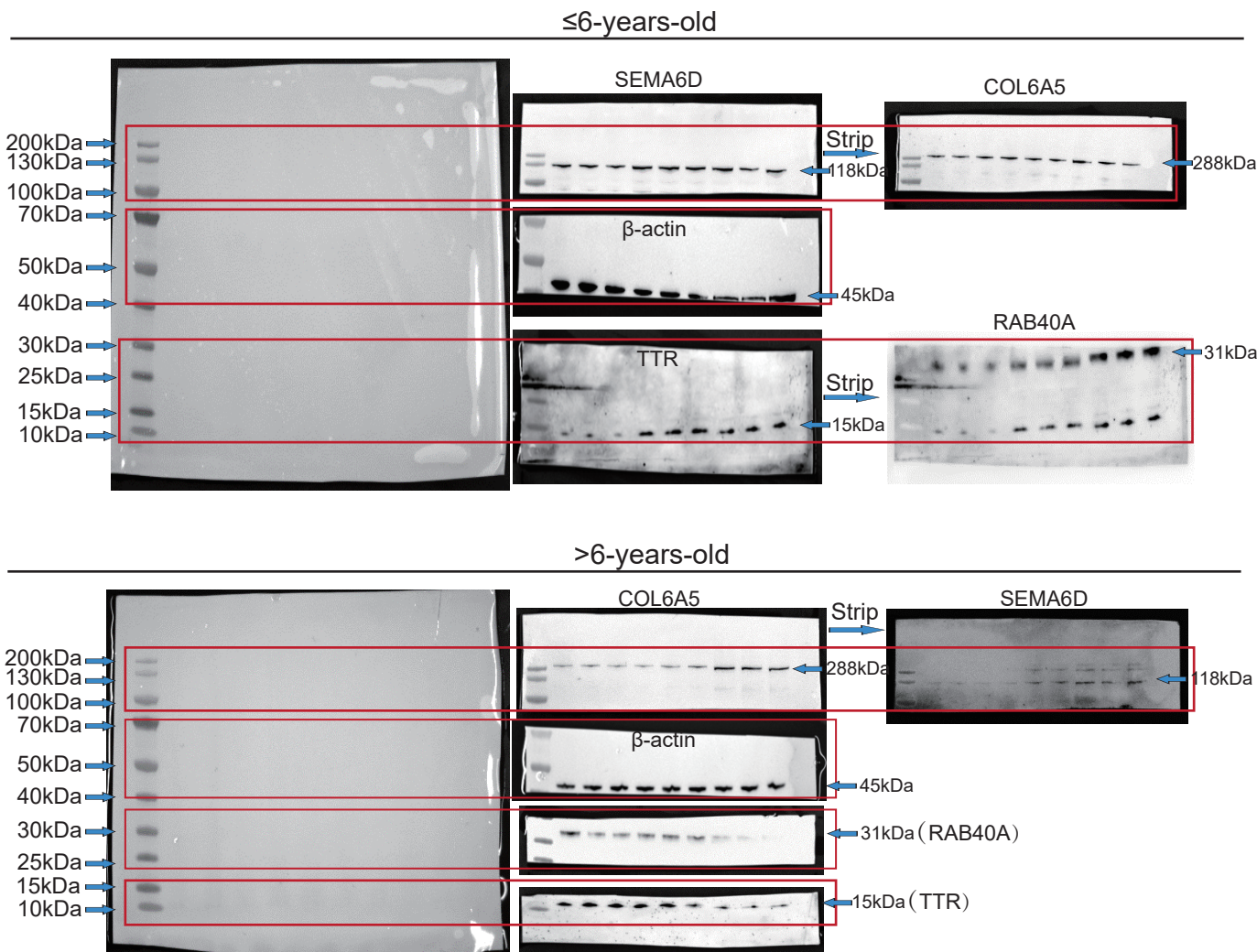

Supplement: Supplementary file 5 [file Image_5.pdf]
